# Gender-dependent miR-375 promoter methylation and the risk of type 2 diabetes

**DOI:** 10.3892/etm.2013.1069

**Published:** 2013-04-18

**Authors:** JIA CHENG, LINGYAN WANG, LEITING XU, HONGWEI WANG, PANPAN LIU, SHIZHONG BU, MENG YE, LINA ZHANG, QINWEN WANG, SHIWEI DUAN

**Affiliations:** 1 Zhejiang Provincial Key Laboratory of Pathophysiology, School of Medicine, Ningbo University, Ningbo, Zhejiang 315211;; 2 Department of Clinical Medicine, Ningbo Kangning Hospital, Ningbo, Zhejiang 315201;; 3 Bank of Blood Products, Ningbo No. 2 Hospital, Ningbo, Zhejiang 315010, P.R. China;; 4 Section of Endocrinology, The University of Chicago Pritzker School of Medicine, Chicago, IL 60637, USA;; 5 Diabetes Center, School of Medicine, Ningbo University, Ningbo, Zhejiang 315211;; 6 Department of Endocrinology, The Affiliated Hospital, School of Medicine, Ningbo University, Ningbo, Zhejiang 315000, P.R. China

**Keywords:** type 2 diabetes, miR-375, DNA methylation, promoter

## Abstract

Promoter DNA methylation may reflect the interaction between genetic background and environmental factors in the development of metabolic disorders, including type 2 diabetes (T2D). As an epigenetic factor of T2D, miR-375 plays an important role in the functional accommodation of islet cells. In the present study, we investigated the association of promoter DNA methylation of the miR-375 gene with the risk of T2D. Using bisulfite pyrosequencing technology, the DNA methylation levels of eight CpG dinucleotides on the miR-375 promoter were measured in 48 T2D cases and 48 healthy controls. The majority of CpGs (with the exception of CpG7) had significantly higher methylation levels in women compared with those in men (P<0.05). The methylation levels of the eight CpGs were significantly correlated with each other (P<0.001). No significant association between miR-375 gene promoter methylation and the risk of T2D was identified (P=0.417). Similar results were observed in the breakdown analysis by gender (men, P=0.844; women, P=0.234). In addition, although a correlation between the CpG8 methylation level of miR-375 and total triglyceride level was identified in women (P=0.009), DNA methylation of the majority of CpGs in the miR-375 gene promoter was not associated with the clinical metabolic features of the individuals.

## Introduction

Diabetes mellitus is a complex metabolic disorder that has become a major public health problem worldwide (1–3). Characterized by insulin resistance, type 2 diabetes (T2D) is the most common subtype of this chronic disease (1,3). Dysfunction of pancreatic β-cells plays a central role in the pathogenesis of diabetes and the individual genetic basis also contributes to the development of this disease (1,4). T2D usually results in numerous complications, including nephropathy, retinopathy and the increased risk of renal failure, blindness and thrombotic disease (1). Although a number of studies have provided valuable information concerning the pathogenesis and clinical treatment of T2D, the current preventive and therapeutic methods are far from satisfactory.

MicroRNAs (miRNAs) are small single-stranded RNA molecules that were first identified in *Caenorhabditis elegans*(5,6). miRNAs are typically 18–26 nucleotides in length and play a crucial role in gene expression by inducing translational arrest and degradation of the mRNAs of target genes (4,7). A number of studies have shown that miRNAs regulate numerous key biological processes, including embryonic development, cellular differentiation, apoptosis and metabolic homeostasis (7–9).

miR-375 is a powerful regulator of pancreatic β-cell function and is essential for normal glucose homeostasis (8,10). miR-375 targets the mRNAs of myotrophin (*Mtpn*), phosphoinositide-dependent protein kinase-1 (PDK-1) and extracellular signal-regulated kinases 1/2 (ERK1/2) to perform physiological and pathological processes, including insulin secretion, the glucose effect and adipocyte differentiation (4,7). miR-375 is abnormally expressed in the serum of Chinese T2D patients (11). However, the precise regulation mechanism of miR-375 expression is poorly understood in diabetes.

Abnormal DNA methylation changes have been shown to be involved in pancreatic β-cell dysfunction and apoptosis (12,13). Peripheral DNA methylation alterations were shown to predict the risk of coronary heart disease (CHD) (14). Blood-based epigenetic diabetes studies have the potential value of forecasting the predisposition to the disease, including T2D (14). The miR-375 gene (chr2:219866367–219866430) is located in the intergenic regions of the *CRYBA2* and *CCDC108* genes (15). This locus and its vicinal area are rich in CpGs that may regulate the expression of the miR-375 gene. In the present study, we hypothesize that the methylation levels of the miR-375 promoter region may contribute to the risk of T2D. We used a non-invasive approach to compare the miR-375 methylation levels of T2D patients with those of gender- and age-matched healthy individuals. This study provides new evidence regarding the identification of epigenetic biomarkers in T2D with a guidance value in prevention and treatment.

## Materials and methods

### Subjects

This study included 48 T2D cases and 48 age- and gender-matched healthy controls who were enrolled at The Affiliated Hospital of Ningbo University (Ningbo, China). The detailed characteristics of the subjects are described in Table I. All individuals were Han Chinese living in Ningbo City for at least three generations. T2D patients were recruited if the plasma glucose levels were >7.0 mmol/l at fasting or >11.1 mmol/l at 2 h after glucose loading (16). All healthy controls were recruited according to the standard of fasting blood glucose <6.1 mmol/l. None of the controls had a family history of T2D in the first degree relatives or had received any medication. Subjects were excluded from this study if they had hypertension, CHD, renal inadequacy, drug abuse or any other serious diseases. Our study was approved by the ethics committee of Ningbo University and written informed consent was obtained from all subjects. Blood samples were collected in 3.2% citrate sodium-treated tubes and then stored at −80°C for DNA extraction.

### Phenotype collection

Blood samples were obtained after a 12 h overnight fast from the antecubital vein into vacutainer tubes containing ethylenediamine tetraacetic acid (EDTA). Plasma levels of cholesterol, triglycerides, alanine aminotransferase (ALT), uric acid and glucose were enzymatically measured using a CX7 Analyzer (Beckman Diagnostics, Fullerton, CA, USA).

### DNA methylation assay

**Figure 1:**
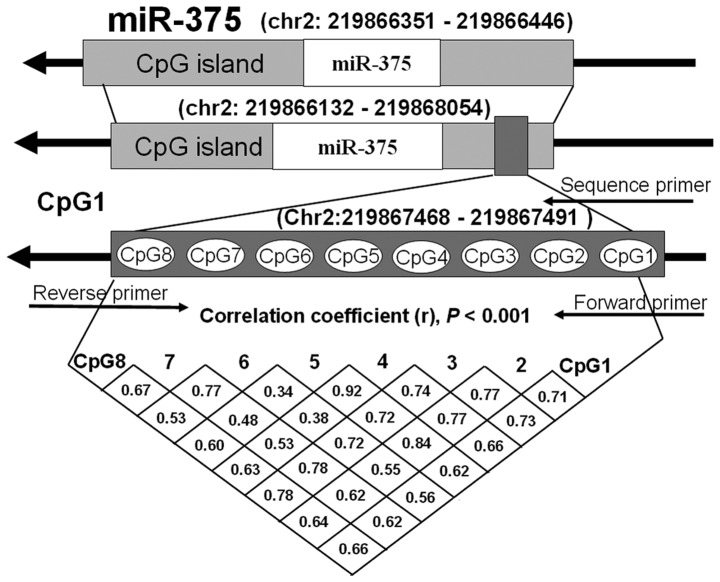
CpG islands on the fragment within the miR-375 promoter.

Human genomic DNA was isolated from peripheral blood samples using the nucleic acid extraction automatic analyzer (Lab-Aid 820, Zeesan, Xiamen, China). DNA was quantified using the PicoGreen^®^ double-strand (dsDNA) DNA Quantification kit (Molecular Probes Inc., Eugene, OR, USA). Bisulfite pyrosequencing technology was used to determine the methylation levels of the eight CpG dinucleotide on the fragment (Chr2:219867468–219867491) within the promoter of the miR-375 gene (Fig. 1). Pyrosequencing assays combined sodium bisulfite DNA conversion chemistry, polymerase chain reaction (PCR) amplification and sequencing by synthesis assay of the target sequence. Sodium bisulfite preferentially deaminated unmethylated cytosine residues to thymines (following PCR amplification), whereas methylcytosines remained unmodified. PCR primers were selected using PyroMark Assay Design software v2.0.1.15 (Qiagen, Germany). The primers used in the PCR and pyrosequencing assay are described in Table II.

### Statistical analysis

Pearson’s Chi-square test was used to compare the categorical variables. Differences in the mean values of continuous variables between the two groups were compared with the Student’s t-test. Using Pearson’s correlation analysis, the associations between miR-375 methylation and metabolic characteristics of T2D subjects were assessed. P<0.05 was considered to indicate a statistically significant difference. All statistical analyses were performed using Statistical Program for Social Sciences (SPSS) software 17.0 (SPSS, Inc., Chicago, IL, USA).

## Results

A total of 96 subjects consisting of 48 T2D cases and 48 ageand gender-matched controls were recruited for the present study. A fragment harboring eight CpG dinucleotides was selected to measure the DNA methylation level of the miR-375 gene promoter (Fig. 1). The clinical characteristics and miR-375 DNA methylation levels of all subjects are shown in Table I. As shown in Table III, the majority of CpGs (with the exception of CpG7) had significantly higher methylation levels in women than in men (P<0.05).

Although CpG6 had a relatively higher level of methylation in the controls than in the T2D cases (Table IV, P=0.034), there were no significant differences in the methylation levels of the other seven CpGs (CpG1-5 and CpG7-8) between the T2D cases and the healthy controls (P>0.05; Table IV). The methylation levels of the eight CpGs were significantly correlated with each other (r>0.30, P<0.001; Fig. 1).

**Figure 2:**
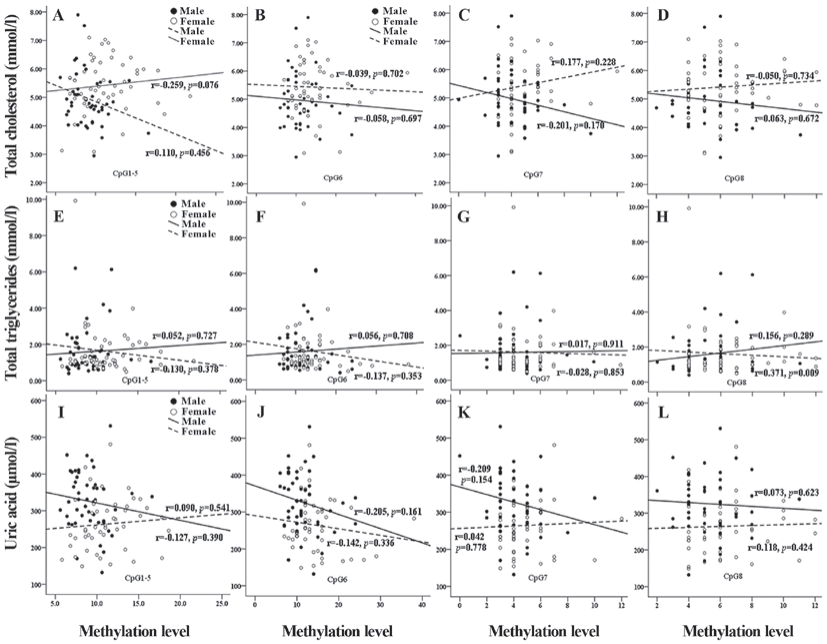
Correlation analysis between the CpG methylation levels of miR-375 and clinical metabolic features.

A breakdown analysis by gender demonstrated that the methylation levels of all eight CpGs were not associated with an increased risk of T2D in men (P>0.10; Table VA). Similar results were observed in women (P>0.05; Table VB), with the exception of a tendency of a higher methylation level of CpG7 (P=0.046; Table V). Furthermore, we analyzed the association between the CpG methylation levels of miR-375 and clinical metabolic features (total cholesterol, total triglycerides and uric acid levels) in the two gender groups. No significant correlation was identified in the above analysis (Fig. 2), with the exception that there was a significant correlation between the CpG8 methylation level of miR-375 and total triglyceride level in women (r=0.371, P=0.009; Fig. 2).

## Discussion

DNA methylation and miRNA expression are important mechanisms of epigenetic regulation in T2D (9–15). A crucial role of miR-375 has been reported in pancreatic islet development (8). However, little is known concerning its methylation status in T2D. In this study, we evaluated the contribution of miR-375 to the risk of T2D.

In the current study, a significant difference in the DNA methylation of miR-375 between male and female subjects was observed (Table III). The majority of the miR-375 CpGs (with the exception of CpG7) demonstrated higher methylation levels in women than in men. This suggests that the methylation regulation mechanism of miR-375 may differ between genders. Previously, estrogen exposure was considered to impact the modulation of the epigenetic modification in the progression of human disease (17–19). The long-term exposure to various types of sex hormone may be one of the causes of this result. In metabolism research, expression of miR-375 has been reported to regulate glucose-induced insulin secretion by targeted silencing of the *Mtpn* gene (7,11,20). Although there was a slight tendency of a higher methylation level of CpG6 in the miR-375 promoter, we identified no significant differences in the the DNA methylation levels of the majority of CpGs between T2D patients and healthy controls (Table IV).

miR-375 is essential for normal glucose homeostasis and adaptive β-cell expansion in insulin resistance (10). Bioinformatic analysis revealed that miR-375 regulates a number of functional genes that control cellular growth and proliferation (10). In order to further identify the epigenetic role of miR-375 in T2D, we performed a case-control comparison of the miR-375 methylation levels in two gender subgroups. Although our results demonstrated that CpG-7 exhibits a relatively higher methylation level in healthy control women than in women with T2D (P=0.046; Table V), there were no other significant differences between healthy controls and T2D patients in the DNA methylation levels of CpGs and T2D across genders (Table V). This phenomenon suggests that there may be no gender-specific role of miR-375 methylation in the process of T2D, and DNA methylation may not be the major mechanism of regulation during its biological function in T2D.

miR-375 has been shown to regulate blood glucose homeostasis and induce adipogenic differentiation, which are associated with the pathophysiological process of T2D (21). Human blood-based aberrant expression of miR-375 was observed in a clinical study (11). The serum miR-375 level in T2D patients was significantly higher in quantitative real-time RT-PCR analysis (11). However, the DNA methylation patterns may act as a tissue-specific feature in disease (22,23). For instance, all the CpG sites of the insulin-2 gene (*Ins2*) promoter presented an unmethylated status in pancreatic islet β-cells; however, they were methylated in other tissues (22,23). A similar tissue-specific methylation pattern was identified in a study of *Ins2* exon 2 (22).

Analysis of candidate gene DNA methylation levels using a case-control study provides knowledge of disease-related mechanisms and is helpful for determining biomarkers of disease and therapy response (24). A number of miRNAs are dysregulated in numerous human organs and tissues during T2D development (7,9). As a member of this family, miR-375 has diverse biological roles in this metabolic disease. However, our results indicated that the methylated CpGs of miR-375 should not be regarded as epigenetic targets in T2D treatment.

In summary, we identified no differential DNA methylation levels of miR-375 between healthy controls and T2D patients; therefore, this type of epigenetic process is not the main regulatory mechanism of miR-375 in T2D. This study provides new information concerning the miRNA epigenetic process which may prove useful in the genetic-based pharmacological development of future T2D treatments.

## Figures and Tables

**Table I:** Characteristics of all subjects (n=96).

Characteristics	Mean ± SE	Range
Age (years)	59.2±7.5	35–69
Gender (M/F)	48/48	
BMI (kg/m^2^)^a^	23.71±3.28	17.15–42.96
Total cholesterol (mmol/l)	5.19±0.96	2.95–7.90
Total triglycerides (mmol/l)	1.60±1.36	0.40–9.92
Glucose (mmol/l)	6.76±2.65	4.38–22.84
ALT (IU/l)	21.5±15.9	5.0–99.0
Uric acid (*μ*mol/l)	294.9±81.1	132.0–531.0
Methylation level (%)		
CpG1	6.81±2.13	2–13
CpG2	6.94±2.73	3–19
CpG3	8.57±2.56	5–20
CpG4	15.35±4.56	5–26
CpG5	13.40±4.78	5–35
CpG6	13.08±5.18	6–38
CpG7	4.49±1.74	0–12
CpG8	5.83±1.99	2–12

a n=86. SE, standard error; M, male; F, female; BMI, body mass index; ALT, alanine aminotransferase.

**Table II:** Primers for miR-375 methylation analysis.

Primer	Sequence
Forward	5′-AGGAGGAGTTGTTGGAGAATATGA-3′
Reverse	5′-Biotin-ACTACCCCCCTAACCCCTCT-3′
Sequencing	5′-GTTTTGAGTGTTTAGGTAAGG-3′

**Table III:** Characteristics of all subjects according to gender (n=96).

Characteristics	Male (n=48)	Female (n=48)	P-value
Age (years)	59.1±8.6	59.4±6.3	0.850
BMI (kg/m^2^)^a^	24.12±4.03	23.37±2.49	0.292
Total cholesterol (mmol/l)	4.96±0.94	5.43±0.93	0.015
Total triglycerides (mmol/l)	1.59±1.28	1.61±1.45	0.959
Glucose (mmol/l)	6.77±3.07	6.76±2.19	0.995
ALT (IU/l)	25.8±20.6	17.3±7.4	0.009
Uric acid (*μ*mol/l)	325.6±79.0	264.3±71.6	0.000
Methylation level (%)			
CpG1	5.88±1.81	7.75±2.04	0.000
CpG2	5.85±1.91	8.02±2.99	0.000
CpG3	7.71±1.95	9.44±2.82	0.001
CpG4	13.98±3.36	16.73±5.18	0.003
CpG5	11.94±3.03	14.85±5.72	0.003
CpG6	11.65±4.21	14.52±5.69	0.006
CpG7	4.17±1.62	4.81±1.79	0.068
CpG8	5.33±1.69	6.33±2.16	0.013

Data are presented as mean ± standard error.

a n=86 (39 male vs. 47 female). BMI, body mass index; ALT, alanine aminotransferase.

**Table IV:** Characteristics of all subjects according to the previous history of diabetes (n=96).

Characteristics	T2D (n=48)	Non-diabetic (n=48)	P-value
Age (years)	59.2±7.5	59.2±7.5	1.000
BMI (kg/m^2^)^a^	24.17±4.18	23.18±1.64	0.146
Total cholesterol (mmol/l)	5.34±0.83	5.05±1.06	0.140
Total triglycerides (mmol/l)	1.90±1.69	1.31±0.82	0.034
Glucose (mmol/l)	8.31±2.91	5.22±0.92	0.000
ALT (IU/l)	25.1±18.5	18.0±12.1	0.028
Uric acid (*μ*mol/l)	289.3±70.5	300.6±90.9	0.499
Average methylation level (%)	9.09±2.07	9.53±3.15	0.417
Methylation level (%)			
CpG1	6.85±1.94	6.77±2.34	0.849
CpG2	6.85±2.32	7.02±3.10	0.766
CpG3	8.35±1.95	8.79±3.06	0.406
CpG4	15.29±3.92	15.42±5.16	0.894
CpG5	13.42±4.35	13.38±5.23	0.966
CpG6	11.96±3.12	14.21±6.48	0.034
CpG7	4.27±1.22	4.71±2.12	0.219
CpG8	5.71±1.83	5.96±2.15	0.542

Data are presented as mean ± standard error.

a n=86 (46 T2D vs. 40 non-diabetic). T2D, type 2 diabetes; BMI, body mass index; ALT, alanine aminotransferase.

**Table V:** Characteristics of all subjects according to gender (n=96).

A. Male (n=48)			

Characteristics	T2D (n=24)	Non-diabetic (n=24)	P-value
Age (years)	59.1±8.7	59.1±8.7	
BMI (kg/m^2^)^a^	24.92±5.17	23.10±1.21	0.124
Total cholesterol (mmol/l)	5.06±0.74	4.86±1.11	0.464
Total triglycerides (mmol/l)	1.81±1.56	1.38±0.90	0.254
Glucose (mmol/l)	8.59±3.49	4.94±0.34	0.000
ALT (IU/l)	30.4±23.8	21.1±15.9	0.119
Uric acid (*μ*mol/l)	304.7±70.6	346.5±82.7	0.066
Average methylation level (%)	8.37±1.80	8.26±2.18	0.844
Methylation level (%)			
CpG1	6.04±1.90	5.71±1.73	0.528
CpG2	6.25±2.05	5.46±1.72	0.154
CpG3	7.83±1.88	7.58±2.04	0.661
CpG4	14.04±3.03	13.92±3.72	0.899
CpG5	12.33±2.91	11.54±3.15	0.371
CpG6	10.79±3.15	12.50±4.97	0.162
CpG7	4.25±1.45	4.08±1.82	0.727
CpG8	5.42±1.77	5.25±1.65	0.737

B. Female (n=48)			

Characteristics	T2D (n=24)	Non-diabetic (n=24)	P-value
Age (years)	59.4±6.4	59.4±6.4	
BMI (kg/m^2^)^b^	23.49±2.97	23.25±1.93	0.747
Total cholesterol (mmol/l)	5.62±0.83	5.24±1.00	0.161
Total triglycerides (mmol/l)	1.98±1.85	1.23±0.75	0.071
Glucose (mmol/l)	8.04±2.22	5.49±1.20	0.000
ALT (IU/l)	19.8±8.6	14.8±4.8	0.019
Uric acid (umol/l)	273.9±68.3	254.6±75.0	0.357
Average methylation level (%)	9.81±2.10	10.81±3.48	0.234
Methylation level (%)			
CpG1	7.67±1.63	7.83±2.4	0.780
CpG2	7.46±2.45	8.58±3.41	0.196
CpG3	8.88±1.92	10.00±3.45	0.171
CpG4	16.54±4.35	16.92±5.99	0.805
CpG5	14.50±5.26	15.21±6.23	0.672
CpG6	13.13±2.66	15.92±7.42	0.094
CpG7	4.29±0.95	5.33±2.26	0.046
CpG8	6.00±1.89	6.67±2.39	0.289

Data are expressed as mean ± standard error.

a n=39 (22 T2D vs. 17 non-diabetic),

b n=47 (24 T2D vs. 23 non-diabetic). T2D, type 2 diabetes; BMI, body mass index; ALT, alanine aminotransferase.

## Abbreviations:

T2D: type 2 diabetes;
ALT: alanine aminotransferase
